# Sequential events during the quiescence to proliferation transition establish patterns of follicle cell differentiation in the *Drosophila* ovary

**DOI:** 10.1242/bio.059625

**Published:** 2023-01-12

**Authors:** Eric H. Lee, Daniel Zinshteyn, Fred Miglo, Melissa Q. Wang, Jessica Reinach, Cindy M. Chau, Joseph M. Grosstephan, Iliana Correa, Kelly Costa, Alberto Vargas, Aminah Johnson, Sheila M. Longo, Jennifer I. Alexander, Alana M. O'Reilly

**Affiliations:** ^1^ Molecular Therapeutics Program, Fox Chase Cancer Center, Philadelphia, PA 19111, USA; ^2^ Immersion Science Program, Fox Chase Cancer Center, Philadelphia, PA 19111, USA; ^3^ Drexel University College of Medicine, Molecular and Cellular Biology and Genetics Graduate Program, Philadelphia, PA 19129, USA

**Keywords:** Stem cell, Self-renewal, Oogenesis, Quiescence to proliferation transition, Cytoplasmic projections, Cell fate, Follicle formation

## Abstract

Stem cells cycle between periods of quiescence and proliferation to promote tissue health. In *Drosophila* ovaries, quiescence to proliferation transitions of follicle stem cells (FSCs) are exquisitely feeding-dependent. Here, we demonstrate feeding-dependent induction of follicle cell differentiation markers, eyes absent (Eya) and castor (Cas) in FSCs, a patterning process that does not depend on proliferation induction. Instead, FSCs extend micron-scale cytoplasmic projections that dictate Eya-Cas patterning. We identify *still life* and *sickie* as necessary and sufficient for FSC projection growth and Eya-Cas induction. Our results suggest that sequential, interdependent events establish long-term differentiation patterns in follicle cell precursors, independently of FSC proliferation induction.

## Teaser

**Summary:** Epithelial follicle stem cells in the fly ovary depend on dynamic, axon-like cytoplasmic projections to re-establish cell fate patterning after a period of nutrient restriction.

## INTRODUCTION

The equilibrium between stem cell self-renewal and differentiation is a cornerstone of tissue health. Heterogeneous stem cell pools must be maintained throughout the lifetime of the animal, while also producing the differentiated daughter cells necessary for optimal tissue function (Goodell and Rando, 2015; Greulich and Simons, 2016). Controlled shifts mediated by changes in signals that promote self-renewal versus differentiation may be leveraged for tissue repair after injury or prevention of aging symptoms (Goodell and Rando, 2015; Xin et al., 2016). In contrast, continuous imbalance can lead to aberrant states such as tumor formation when self-renewal is favored, or stem cell loss when differentiation is the primary outcome. Defining the molecular mechanisms that determine stem cell fate is therefore a pressing need.

Housed in microenvironments called niches, stem cells rely on their surroundings for signals and nutrients that enable self-renewal and differentiation (Xin et al., 2016). In cases like the well-studied germline stem cells (GSCs) in *Drosophila*, signals from the niche confer near-immortal status, ensuring long-term functional lifespan of individual GSC clones and inheritance of stem cell function through generations (Hinnant et al., 2020). Other stem cells, including epithelial follicle stem cells (FSCs) in the fly ovary, exist in an aggressive, competitive environment, where limited niche space drives selection of stem cells in the right time and place to self-renew, with losers of the competition displaced to undergo differentiation (Albert Hubbard and Schedl, 2019; Clevers and Watt, 2018; Nelson et al., 2019; Rust and Nystul, 2020).

Recent evidence points to proliferation rates as key for competitive edge in stem cell niches, with higher rates of proliferation associated with retention (Amoyel et al., 2014; de Navascués et al., 2012; Greulich and Simons, 2016; Hsu et al., 2017; Jin et al., 2008; Kronen et al., 2014; Reilein et al., 2018; Snippert et al., 2010; Su et al., 2018). Over time, stem cells with even a slight proliferative advantage can take over the niche, resulting in a clonal stem cell population and elimination of the initial heterogenous pool (Greulich and Simons, 2016). This drift toward clonality is associated with loss of stem cell function and consequent tissue aging in multiple stem cell populations, with significant work focused on developing strategic approaches that maintain heterogeneity to promote healthy aging (Haas et al., 2018; Wahlestedt et al., 2017). Emerging evidence suggests that imposing ‘quiescent’ resting states equalizes stem cells within a pool, reducing the effects of proliferative advantage and promoting fair competition upon re-initiation of proliferation (Cho et al., 2019; Greulich and Simons, 2016; Urbán et al., 2019; Urbán and Cheung, 2021; van Velthoven and Rando, 2019). In quiescence, stem cells exit the cell cycle and remain poised between mitosis and G1 in a reversible state also known as G0 (Cho et al., 2019). Stimuli such as feeding, injury, or other signals trigger G1 entry and progression to a proliferative stem cell state (Novak et al., 2021; Urbán and Cheung, 2021). In some stem cell populations, nutrient restriction promotes quiescence, with alternating periods of fasting and feeding controlling reversible quiescence to proliferation (Q→P) shifts to maintain heterogeneity through the aging process (Bruens et al., 2020; Hartman et al., 2013; Schultz and Sinclair, 2016; Spehar et al., 2020; van Velthoven and Rando, 2019). The ability to manipulate stem cell pools through diet presents an opportunity to define cellular processes involved in Q→P transitions and to uncover molecular mechanisms that may uncover intervention strategies that promote healthy aging.

**Fig. 1.**
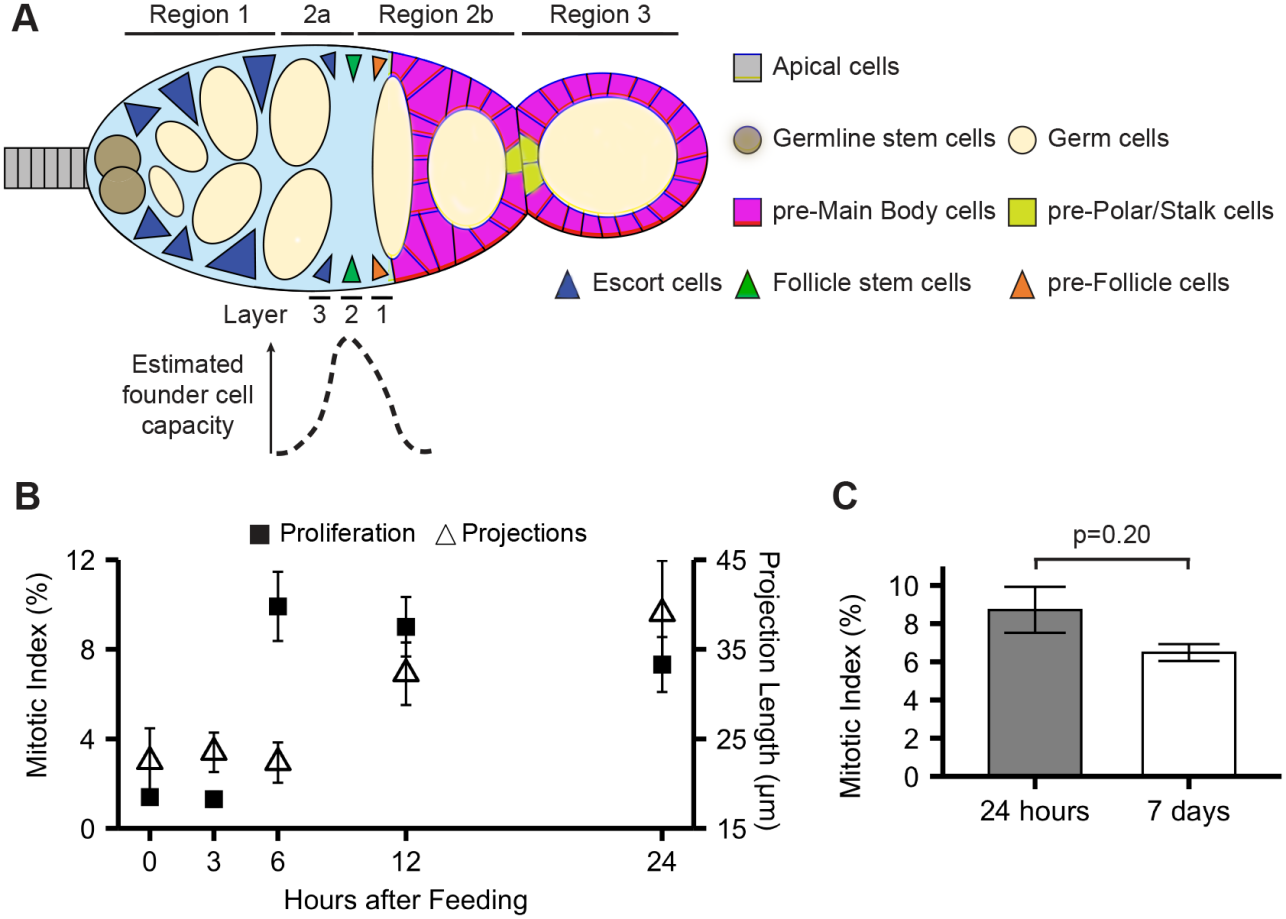
**Establishment of steady-state patterns of proliferation and projection extension in the first 24 h after re-feeding.** (A) Schematic diagram of the germarium. FSCs (green) are located at the Region 2A/2B border/Layer 2. Layer 1 (orange) and Layer 3 (blue) cells preferentially produce follicle cells (magenta) or inner germarial sheath (IGS/escort) cells (blue), respectively, but have the capacity to function as FSCs. Germline cysts (cream), interact with FSCs and become encapsulated by follicle cells to form egg chambers. Differentiation into Polar/Stalk cell precursors (pre-PS, gold) occurs at the anterior and posterior poles of each egg chamber, with main body cell precursors (pre-MB, magenta) surrounding germline cysts. Apical cells (gray), germline stem cells (brown), and IGS/escort cells (blue) reside in Region 1. Estimated founder cell capacity in Layers 3, 2, 1 is shown (bottom), with the peak of ‘stemness’ in Layer 2 (Hayashi et al., 2020; Melamed and Kalderon, 2020; Reilein et al., 2017; Waghmare and Page-McCaw, 2018). (B) Time course of proliferation and projection extension. Timepoints at 0 (nutrient-restricted), 3, 6, 12, and 24 h after re-feeding are shown. Mitotic index is indicated as the frequency of germaria with a Layer 2 FSC in mitosis (PH3+). Projection length (μm) in MARCM-CD8-GFP-labeled Layers 2 and 1 cells is shown at indicated timepoints. Proliferation was assayed in *109-30-Gal4TubGal80^ts^* control flies (*N*=571, 548, 435, 427, 509 for 0, 3, 6, 12, and 24 h after re-feeding, respectively); projections were assayed from mosaic clones generated from MARCM stocks (*N*=10, 10, 9, 11, 6 for 0, 3, 6, 12, and 24 h after re-feeding timepoints, respectively). Plots represent mean±s.e.m. (C) Mitotic index of layer 2 FSCs, 24 h (*N*=553) and under steady-state conditions, 7 days after re-feeding (*N*=444); genotype=*109-30-Gal4TubGal80^ts^*/+. *P*=0.20, χ^2^ test. Plot represents mean±s.e.m.

Stem cells that belong to the competitive stem cell paradigm, including FSCs, are exquisitely feeding-dependent, undergo Q→P transitions, use proliferative advantage for long-term retention, and drift toward clonality over time (Drummond-Barbosa and Spradling, 2001; Greulich and Simons, 2016; Hartman et al., 2013; Hsu et al., 2017; Kirilly et al., 2005; Kronen et al., 2014; Reilein et al., 2018; Snippert et al., 2010; Song and Xie, 2003; Su et al., 2018; Wang and Page-McCaw, 2014; Wang et al., 2012; Wang and Kalderon, 2009). Hedgehog (Hh) signaling translates feeding status to control FSC Q→P transitions (Hartman et al., 2013). Specifically, in response to cholesterol ingestion, Hh is released from terminal filament and cap cells (apical cells) in the stem cell compartment of the fly, called the germarium (Fig. 1A) (Çiçek et al., 2016; Hartman et al., 2013). Hh accumulation correlates precisely with proliferation induction within FSCs (Hartman et al., 2013), which then undergo self-renewal and/or initiate differentiation into epithelial follicle cells (Margolis and Spradling, 1995; Nystul and Spradling, 2007; Reilein et al., 2017, 2018).

Location within the germarium is a primary predictor of fate for cells with FSC potential. Cells located at the Region 2A/2B border (also called Layer 2) have the highest propensity to self-renew and are notably feeding-responsive (Fig. 1A) (Dai et al., 2017; Hartman et al., 2013, 2015; Margolis and Spradling, 1995; Nystul and Spradling, 2007; Reilein et al., 2017, 2018). Cells in Region 2A, one cell diameter to the anterior (Inner Germarial Sheath, IGS/escort cells/Layer 3) or posterior in Region 2B (Layer 1) also are capable of self-renewal, but exhibit a strong preference for differentiation into escort cells or follicle cells, respectively (Melamed and Kalderon, 2020; Reilein et al., 2017; Rust et al., 2020) (Fig. 1A). Layer 3 cells do not divide in response to feeding, emphasizing the key function of Layer 2 cells as founders of the follicular epithelium following a period of nutrient restriction (Hartman et al., 2013). Differentiating follicle cell daughters generated by FSCs encapsulate 16-cell germline cysts, forming follicles (egg chambers) comprised of a single-layered cuboidal epithelium and a 16-cell germline cyst that develop synchronously through 14 stages of development to produce a mature egg (Fig. 1A). Within the FSC pool, divisions are asynchronous, often with only one FSC dividing at a time (Melamed and Kalderon, 2020; Reilein et al., 2017). Cells residing in Region 2A-B can differentiate into follicle cells without division (Melamed and Kalderon, 2020; Reilein et al., 2018), suggesting that multiple mechanisms are employed to maintain a long-lived stem cell pool and produce sufficient functional daughter cells. Recent work demonstrates overlapping gene expression signatures and the ability to change position among cells in and near the FSC niche (Jevitt et al., 2020; Reilein et al., 2018; Rust et al., 2020; Slaidina et al., 2020, 2021; Tu et al., 2021), indicating plasticity among cellular residents in Region 2A-B. Despite these advances, the relationships between cell cycle entry, dynamic changes in morphology and position, and self-renewal versus differentiation fate decisions of FSCs are not well understood.

Here, we took advantage of the ability to stimulate FSC Q→P transitions via feeding to ascertain the sequence of events that establishes self-renewal-differentiation patterning. Interestingly, we find that the Q→P transition has little influence on induction of differentiation patterns upon feeding. Instead, feeding-dependent growth of micron-scale cytoplasmic projections extended by FSCs precedes differentiation patterning, with key regulators of FSC projection outgrowth, *still life* (*sif*) and *sickie* (*sick*), necessary and sufficient for both events. Our results support a model in which feeding induces a step-wise sequence of events that influence FSC outcomes independently (e.g. proliferation) and interdependently (e.g. projection growth and differentiation patterning).

## RESULTS

### Feeding drives FSC proliferation and differentiation

FSCs enter a non-proliferative, quiescent state when flies are raised on grape juice plates (Hartman et al., 2013). Grape juice plates provide water and simple sugars to sustain life, but lack the protein and complex nutrients required for egg production. Feeding nutrient-restricted flies (referred to as 0 h throughout this work) with yeast-rich food (referred to as re-feeding) rapidly stimulates FSCs out of quiescence (Hartman et al., 2013). The initial peak of proliferation occurs 6 h after re-feeding, resolving to a steady-state rate by 24 h that is maintained long-term, for at least 7 days after re-feeding (Fig. 1B,C).

A primary function of FSCs is to generate daughter cells that differentiate to form ‘main body’ cells of the follicular epithelium, as well as subsets of follicle cells known as polar and stalk cells (Bai and Montell, 2002; Chang et al., 2013; Dai et al., 2017; Margolis and Spradling, 1995; Nystul and Spradling, 2007; Reilein et al., 2017, 2018; Tworoger et al., 1999). Upon exiting Layer 1 of the germarium, cells initiate differentiation into main body follicle cell precursors (pre-MBs), which surround developing germline cysts, or polar or stalk cell precursors (pre-PS) located at the posterior and anterior poles (Fig. 1A). Stalk cells link adjacent egg chambers within individual ovarioles, and polar cells produce factors that control signaling gradients to dictate cell fate (Baksa et al., 2002; Borensztejn et al., 2018; Chang et al., 2013; Ghiglione et al., 2002; Grammont and Irvine, 2002; McGregor et al., 2002). Entry into the differentiation program is characterized by robust upregulation of the adhesion molecule, Fasciclin III (FasIII, Zhang and Kalderon, 2000) as well as two transcription factors, eyes absent (Eya) and castor (Cas), that serve as markers for differentiation status and function (Bai and Montell, 2002; Chang et al., 2013; Dai et al., 2017). Cells that lack Eya and express high Cas (Eya^−^, Cas^+^) adopt fully differentiated polar/stalk cell fates, with cells expressing high Eya and no Cas (Eya^+^, Cas^−^) differentiating as main body follicle cells.

**Fig. 2.**
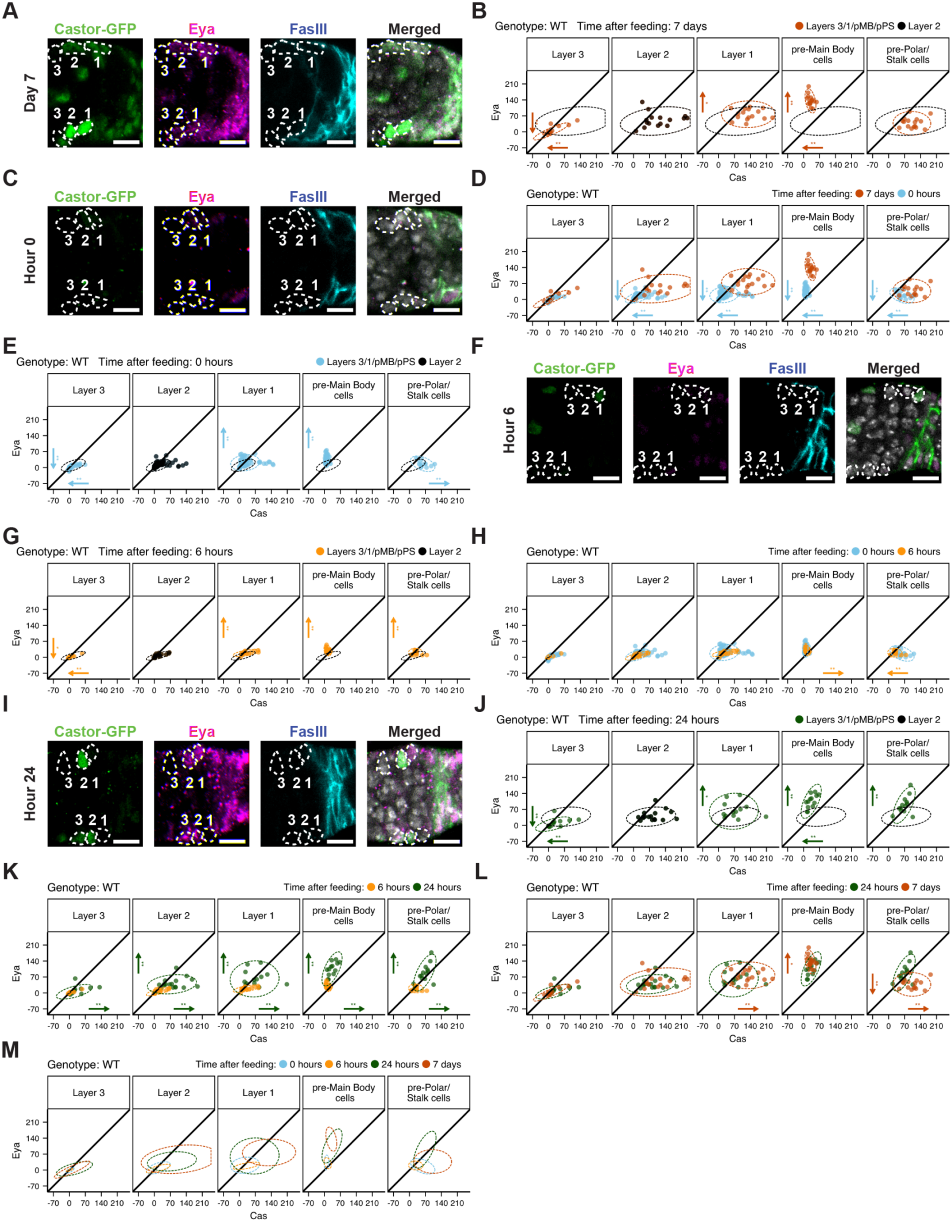
**Differentiation signatures are feeding dependent.** (A) Representative images demonstrating Eya (magenta), Castor-GFP (green), and FasIII (blue, follicle cells) expression in germaria fed for 7 days after a period of nutrient restriction, plus nuclei (DRAQ5, white). Layers 3, 2, 1 are circled (white dashes). (B) Background-subtracted mean fluorescence intensities (MFI), with each dot representing the MFI of a single cell in Layers 3, 2, 1, pre-MB or pre-PS cells (*N*=16). Dotted lines are data ellipses drawn around the specified cells at each layer, representing 95% of the distribution. Layer 2 FSCs are drawn in black; Layer 2 distribution is shown at each layer for comparison. Vertical (Eya) and horizontal (Cas) arrows indicate significant changes (*P<0.05,**P<0.01) relative to Layer 2, pointing in the direction of change. (C) Representative images demonstrating Eya (magenta), Castor-GFP (green), and FasIII (blue, follicle cells) expression in germaria nutrient-restricted for 3 days, plus nuclei (DRAQ5, white). Layers 3, 2, 1 are circled (white dashes). (D) MFI of single cells in Layers 3, 2,1, pre-MB or pre-PS cells in flies nutrient-restricted for three days (*N*=62). Comparisons between 7 days after re-feeding (orange) and nutrient-restricted flies (blue) show Eya-Cas signatures in Layers 2, 1 that resemble a more anterior/Layer 3 fate upon nutrient restriction. Vertical (Eya) and horizontal (Cas) arrows indicate significant changes (*P<0.05,**P<0.01) in nutrient-restricted cells relative to the same cell types in steady-state (7 days after re-feeding), pointing in the direction of change. (E) Comparison of MFIs of each layer (blue) relative to Layer 2 (black) demonstrates substantial overlap in nutrient-restricted flies. Vertical (Eya) and horizontal (Cas) arrows indicate significant changes (*P<0.05,**P<0.01) relative to Layer 2, pointing in the direction of change. (F) Representative images demonstrating Eya (magenta), Castor-GFP (green), and FasIII (blue, follicle cells) expression in germaria fed for 6 h after a period of nutrient restriction, plus nuclei (DRAQ5, white). Layers 3, 2, 1 are circled (white dashes). (G) Comparison of MFIs of each layer (yellow) relative to Layer 2 (black) demonstrates substantial overlap 6 h after re-feeding (*N*=16), similar to nutrient-restricted flies. Vertical (Eya) and horizontal (Cas) arrows indicate significant changes (*P<0.05,**P<0.01) relative to Layer 2, pointing in the direction of change. (H) Comparison of MFIs of nutrient-restricted flies (blue) relative to 6 h re-fed (yellow) indicates no change in Layer 3,2,1 Eya-Cas signatures relative to nutrient restriction. Vertical (Eya) and horizontal (Cas) arrows indicate significant changes (*P<0.05,**P<0.01) relative to 0 h, pointing in the direction of change. (I) Representative images demonstrating Eya (magenta), Castor-GFP (green), and FasIII (blue, follicle cells) expression in germaria fed for 24 h after a period of nutrient restriction, plus nuclei (DRAQ5, white). Layers 3, 2, 1 are circled (white dashes). (J) Comparison of MFIs of each layer (green) relative to Layer 2 (black) demonstrates distinctions between layers 24 h after re-feeding. Vertical (Eya) and horizontal (Cas) arrows indicate significant changes (*P<0.05,**P<0.01) relative to Layer 2, pointing in the direction of change. (K) Comparison of MFIs of flies re-fed for 6 h (yellow) relative to 24 h re-fed (green) demonstrates highly significant posterior shifts in Eya-Cas signatures in all layers. Vertical (Eya) and horizontal (Cas) arrows indicate significant changes (*P<0.05,**P<0.01) relative to 6 h, pointing in the direction of change. (L) Comparison of MFIs of flies re-fed for 24 h (green) relative to 7 days re-fed (orange) demonstrates that Eya-Cas signatures in Layers 3, 2 are established by 24 h after feeding. In contrast, significant changes are observed between 24 h and 7 days after re-feeding in Layer 1, pre-PS, and pre-MB cells. Vertical (Eya) and horizontal (Cas) arrows indicate significant changes (*P<0.05,**P<0.01) relative to 24 h, pointing in the direction of change. (M) Data ellipses of MFIs across all cell layers during the Q→P transition and steady-state indicate time-dependent changes in Eya-Cas expression upon re-feeding. (A-D) Scale bars: 10 μm. (A,C,F,I) Eya and FasIII intensities were uniformly increased for improved visibility. Genotype (“WT”, all panels)= *109-30-Gal4TubGal80ts*/+; *Cas::GFP*/+. Unpaired Mann–Whitney tests with Benjamini Hochberg correction for multiple samples were used for (B,D,E,G,H,J-L).

To establish baseline Eya-Cas ‘signatures’ that reflect this documented continuum of functional plasticity in steady-state feeding conditions, we quantified Eya and Cas levels (Dai et al., 2017) (Fig. 2A,B). Eya-Cas expression patterns in pre-MB (Eya^hi^, Cas^lo^) and pre-PS cells (Eya^lo^, Cas^hi^) reflected final differentiation outcomes (Fig. 2B). Some expression of both markers was observed in each precursor cell population, supporting prior work indicating that follicle cells in the germarium have not yet reached fully differentiated status (Bai and Montell, 2002; Chang et al., 2013; Dai et al., 2017). Importantly, pre-MB cells were entirely separable from pre-PS cells within a 95% sample distribution (Fig. 2B), enabling straightforward interpretation of differentiation status and establishing a quantitative baseline for delineating cell fate changes upon genetic or feeding-based manipulation.

**Fig. 3.**
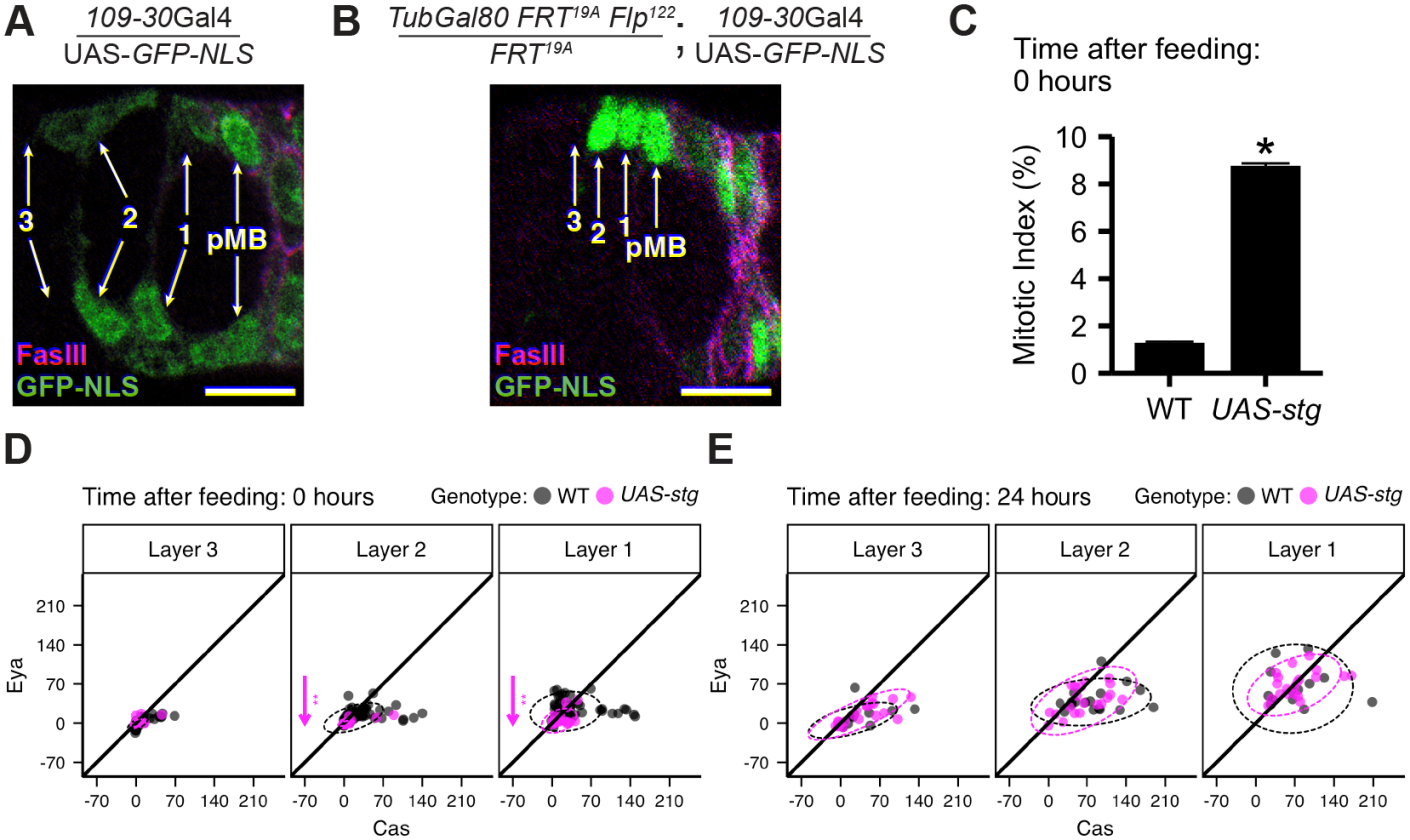
**Proliferation and differentiation induction are independent events.** (A) Nuclear-localized GFP (GFP-nls, green) expression driven by *109-30* Gal4. GFP is expressed in Layers 2, 1, pre-MB and pre-PS cells (arrows, pre-MB/pre-PS cells=magenta). (B) MARCM labeling with *109-30-Gal4* driving GFP-nls (green) indicates Layer 2 cells as founders of the follicular epithelium. FasIII (magenta) and Layers 3, 2, 1, and pre-MB cells are indicated (arrows). (C) Mitotic index of Layer 2 FSCs in wild-type (WT, *109-30-Gal4 TubGal80^ts^*/+, *n*=538) versus *string* overexpressing FSCs (*109-30-Gal4 TubGal80^ts^*/*UAS-string*, *n*=507) in nutrient-restricted conditions. *P<0.01 versus WT, χ^2^ test. (D,E) MFI of FSC niche images from germaria 0 h (nutrient-restricted, D) and 24 h (E) after re-feeding (black (WT)=*109-30-Gal4 TubGal80^ts^*/+; *Cas::GFP*/+, magenta=*109-30-Gal4 TubGal80^ts^*/*UAS-string*; *Cas::GFP*/+). Each dot represents the MFI of a single cell; data ellipses represent 95% of the distribution. Arrows indicate significant [*P<0.05,**P<0.01 (unpaired Mann–Whitney test with Benjamini Hochberg correction for multiple samples] changes in Eya or Cas expression relative to WT. Left to right: (D) *n*=62, 20; (E) *n*=17, 20. Plots represent mean±s.e.m.

We next compared Eya-Cas expression in germarium cells. To focus our attention on cells with FSC potential, we used three complementary criteria: (1) location, (2) lineage labeling, and (3) marker expression. In terms of location, Layer 1 cells are located immediately anterior to strong, differentiated FasIII expressing cells (Fig. 1A). Layer 2 cells are located one cell diameter further to the anterior, and Layer 3 cells reside two cell diameters anterior to the FasIII border (Fig. 1A, (Melamed and Kalderon, 2020). For lineage labeling, the *109-30-*Gal4 transcriptional activator, which we previously showed drives expression in Layer 2, 1, pre-MB, pre-PS, polar and stalk cells (Fig. 3A) (Hartman et al., 2013, 2015) is a valuable tool. The anterior-most founder cells of the follicular epithelium, most frequently located in Layer 2, can be lineage labeled by combining *109-30*-Gal4 with the MARCM system, enabling induction of GFP in mitotically active cells that express *109-30*-Gal4 after a brief heat shock (Singh et al., 2018) (Fig. 3B). In addition to these traditional approaches, use of protein expression-based metrics is beneficial. To date, FSCs have been identified based on location at the Region 2A/2B border of the germarium (‘Region system’). The Region system depends on the relative placement of follicle cell precursors to germline cysts in and near the FSC niche. This relationship changes as germline cysts approach, flatten, and pass through the FSC niche, as well as upon egg chamber budding. The geographical complexities and contentious interpretation of lineage tracing studies have led to substantial controversy and confusion (Fadiga and Nystul, 2019; Kalderon, 2022; Rust et al., 2020). By contrast, FasIII is expressed at high levels only in differentiating pre-follicle cells, with a clearly visible boundary between pre-MB/pre-PS cells and Layer 1 that makes it an excellent landmark for the analysis.

Using the Region system, prior work established a quantitative approach that links levels of Eya and Cas with cell fate, such that Region 2A cells (IGS/escort cells) generally do not express either marker, FSCs at the Region 2A/2B border are marked by low, equal expression of both markers, and cells in Region 2B exhibit a significant, but still equal, increase in Eya and Cas expression (Dai et al., 2017). These expression patterns correlate with FSC founder cell character reported for cells in each layer, with a U-curve of stemness peaking at Layer 2 (Fig. 1A, (Melamed and Kalderon, 2020; Reilein et al., 2017, 2018).

In agreement with prior work, we found that Eya exhibited graded expression that was low/undetectable in Layer 3 IGS/escort cells, (Fig. 2A,B), increased in Layer 2 FSCs, and increased again robustly in Layer 1 (Fig. 2A,B) after 7 days of continuous feeding. Cas exhibited substantially more variability between individual germaria, with some exhibiting high expression in a single layer and no expression elsewhere, graded expression, or other patterns (Fig. 2A,B). The breadth of Cas expression differences were most evident in Layers 2 and 1, where the full measurable range of Cas levels was observed (Fig. 2B). These data suggest that cells in Layers 2 and 1 may span the continuum of Cas expression to provide adequate plasticity for generation of daughter cells with multiple distinct fates. For example, Cas^hi^ Layer 2 and 1 cells may be predisposed to adopt pre-MB or pre-PS fates, whereas cells with Cas levels that approach a 1:1 Eya:Cas ratio may be more likely to self-renew (Dai et al., 2017). Moreover, these data emphasize notable heterogeneity in Eya-Cas signatures among cells in these layers (Fig. 2B).

We used the Layer 2 signature as a baseline for comparison of Eya-Cas signatures between Layers and at timepoints after re-feeding as these cells are (1) responsive to feeding and (2) most likely to serve as founders of the follicular epithelium upon re-feeding nutrient-restricted flies (Hartman et al., 2015; Melamed and Kalderon, 2020; Reilein et al., 2017, 2018). Notable overlap was observed in the Eya-Cas signatures in Layers 2 and 1 in steady-state feeding conditions, measured in 7 day old flies (Fig. 2B). This is expected, as both cell types can function as FSCs. Layer 1 cells exhibited significantly higher levels of Eya (Fig. 2A,B), consistent with reports that Layer 1 cells have a higher propensity to differentiate. Cells in Layer 3 exhibited a mostly distinct signature relative to Layer 2 in steady-state feeding conditions, with significantly lower levels of Eya and Cas (Fig. 2A,B). These results confirm prior results demonstrating that Eya-Cas are reporters of differential FSC potential among cells in the germarium and establish steady-state signatures for each layer for measurement of impacts of genetic or environmental changes upon these cell fate markers.

Dramatic changes in Eya-Cas expression were observed upon nutrient restriction (‘0 hours’, Fig. 2C,D). Expression of both Eya and Cas dropped significantly relative to steady-state feeding levels (‘7 days’ of continuous feeding after a period of nutrient restriction) in all cell types examined except Layer 3, which had extremely low expression even in fed conditions (Fig. 2C,D). Whereas Eya-Cas signatures were location-dependent under steady-state feeding conditions (Fig. 2B), nutrient restriction effectively neutralized major differences, with all cell fates shifting anterior toward a more Layer 3-like signature (Fig. 2D). Although some distinctions remained, Eya-Cas patterns in nutrient-restricted Layer 1 and 3 cells overlapped extensively with the Layer 2 nutrient-restriction signature [Fig. 2E, Layer 2 (black) versus Layers 3 or 1(blue)], suggesting that nutrient restriction promotes some degree of equilibration between cells with FSC potential. The relative differences in Eya-Cas signatures between Layers with FSC potential were maintained 6 h after re-feeding (Fig. 2F,G), when proliferation peaks in Layer 2 FSCs (Fig. 1B, (Hartman et al., 2013). No new induction of Eya or Cas was observed at this timepoint in Layers 3, 2, 1 [Fig. 2H, 0 h (blue trace) and 6 h (yellow trace) are indistinguishable]. By 24 h after re-feeding, however, distinct Eya-Cas patterns relative to Layer 2 FSCs were observed (Fig. 2I,J), and dramatic increases in both markers were observed relative to 6 h after re-feeding (Fig. 2K). Moreover, Eya-Cas expression levels in Layers 2 and 3 were indistinguishable from steady-state levels measured 7 days after re-feeding, [Fig. 2L, 24 h (green) versus 7 days (orange)], suggesting that differentiation marker patterning is established between 6 and 24 h after re-feeding. By contrast, Layer 1 cells, as well as pre-MB and pre-PS cells, did not reach steady-state Eya-Cas patterning by 24 h after re-feeding, despite dramatic increases in Eya-Cas expression between 6 and 24 h (Fig. 2K,L). This observed refinement in Eya-Cas patterning between 24 h and 7 days may (1) depend on time-dependent induction of signaling pathways that drive differentiation patterning (e.g. JAK-STAT) (Baksa et al., 2002; Borensztejn et al., 2018; Chang et al., 2013; Ghiglione et al., 2002; Grammont and Irvine, 2002; McGregor et al., 2002), (2) occur only in FSC daughters that experience continuous feeding, with cells exposed to nutrient restriction retaining more plasticity than steady-state fed counterparts, or (3) reflect a pulse of Eya and/or Cas expression that establishes ‘differentiation memory’ where pre-MB, pre-PS, and pre-follicle cells that alter their Eya-Cas levels upon nutrient restriction received sufficient signaling information to proceed along their fated paths, despite failing to achieve steady-state Eya-Cas levels after re-feeding.

Given the central role of Layer 2 FSCs as founders of the follicular epithelium after feeding, we focused on understanding how the sequential processes induced by re-feeding impact Layer 2 FSCs in particular. Our timecourse analysis demonstrated that proliferation induction in Layer 2 FSCs precedes differentiation patterning temporally (Figs 1B and 2M). One possibility is that induction of proliferation triggers a series of events that instruct differentiation patterning. To test this, we first asked whether simply inducing FSC proliferation is sufficient to induce Eya-Cas expression after a period of nutrient restriction. Proliferation was ectopically induced in FSCs by expressing the CDC25 homolog, String (Edgar and Datar, 1996; Edgar and O'Farrell, 1989). String dephosphorylates Wee1 kinase to drive entry into M phase of the cell cycle, effectively bypassing upstream signals to induce proliferation (Dunphy and Kumagai, 1991). String was expressed in Layer 2, 1, pre-MB and pre-PS cells under control of *109-30*-Gal4 (Fig. 3A,B). Expression of String was sufficient to induce robust activation of proliferation under nutrient restriction (Fig. 3C). By contrast, Eya and Cas expression was not induced in proliferating, String-expressing FSCs (Fig. 3D). In fact, *string* expression under nutrient restriction further reduced Eya expression slightly in Layers 1 and 2 (Fig. 3D). Overexpression of *string* 24 h after re-feeding had no effect on Eya or Cas expression in Layers 3, 2, or 1 (Fig. 3E), suggesting the observed mild suppressive effects may occur only under nutrient restriction. This evidence suggests that differentiation patterning is not a requisite result of proliferation induction, but requires additional or distinct feeding-dependent signals.

### Timecourse of FSC projection dynamics

**Fig. 4.**
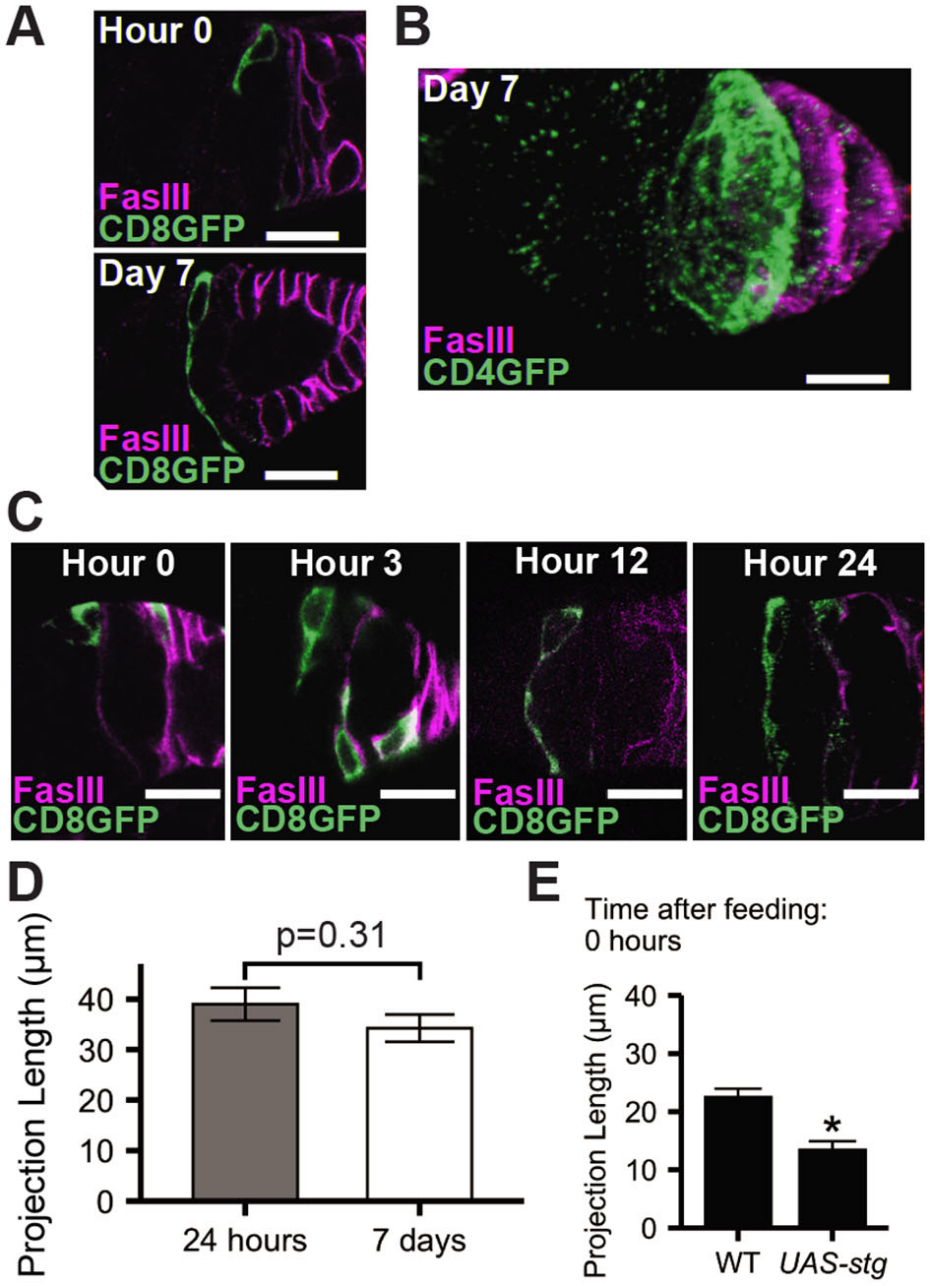
**FSC projection length is established between 12 and 24 h after re-feeding.** (A) MARCM-labeled (*Ub-RFP, Gal80 FRT^19A^ Flp^122^*/*FRT^19A^*; *109-30-Gal4*/*UAS-CD8-GFP*, green) projections in nutrient-restricted or continuously fed Layer 2 FSCs with FasIII (magenta). (B) Three-dimensional image of confocal stack of MARCM-labeled FSCs (green, CD4-GFP) with FasIII (magenta). (C) Timecourse of MARCM-labeled (CD8-GFP, green) FSC projections at 0, 6, 12, and 24 h. (D,E) Average projection length of cells in Layers 2, 1. (D) 24 h (*N*=6) versus 7 days (*N*=6) after re-feeding; genotype=*Ub-RFP, Gal80 FRT^19A^ Flp^122^*/*FRT^19A^*; *109-30-Gal4*/+. *P*=0.30 (n.s.), unpaired Mann–Whitney *U*-test. (E) Time 0 (nutrient-restriction) FSCs overexpressing *string* (*UAS-string*, *Ub-RFP, Gal80 FRT^19A^ Flp^122^*/*FRT^19A^*; *109-30-Gal4*/*UAS-CD8-GFP*; *UAS-string*/+) versus WT (*Ub-RFP, Gal80 FRT^19A^ Flp^122^*/*FRT^19A^*; *109-30-Gal4*/+). *P<0.01, unpaired Mann–Whitney *U*-test. (D,E) Plots represent mean±s.e.m.

We previously reported that FSCs undergo dramatic morphological changes upon feeding (Hartman et al., 2015). In nutrient-restricted conditions, FSCs reside at the surface of the germarium and extend short, microtubule-containing cytoplasmic projections along the basement membrane (Hartman et al., 2015) (Fig. 4A). Upon re-feeding, these projections undergo significant growth, more than doubling in length and extending fully across the germarium (Hartman et al., 2015) (Fig. 4A). Extension of projections from all FSCs residing around the circumference of the germarium results in construction of a web-like network that spans the FSC niche at the Region 2A/2B border (Hartman et al., 2015) (Fig. 4B). As the function of FSC projections and their relationship to proliferation induction and establishment of differentiation patterning are unclear, we first conducted a timecourse of proliferation and projection growth (Fig. 1B). Whereas the proliferation peak was observed at 6 h after re-feeding, no significant change in projection length was observed until the 12-h timepoint (Figs 1B and 4C), making it unlikely that projection growth stimulates proliferation. Projection length was maintained in steady-state conditions (Fig. 4D, 7-days), supporting the idea that the first 24 h after re-feeding is a critical period for establishing patterns that promote long-term FSC function. Overexpression of *string* was not sufficient to induce projection growth in nutrient-restricted FSCs and in fact resulted in shorter projections (Fig. 4E), mirroring the effects of *string* overexpression on Eya-Cas patterning (Fig. 3D). Thus, feeding-dependent projection growth occurs temporally between proliferation induction at 6 h and differentiation patterning at 24 h.

### Sif/TIAM-1 regulates FSC projections

**Fig. 5.**
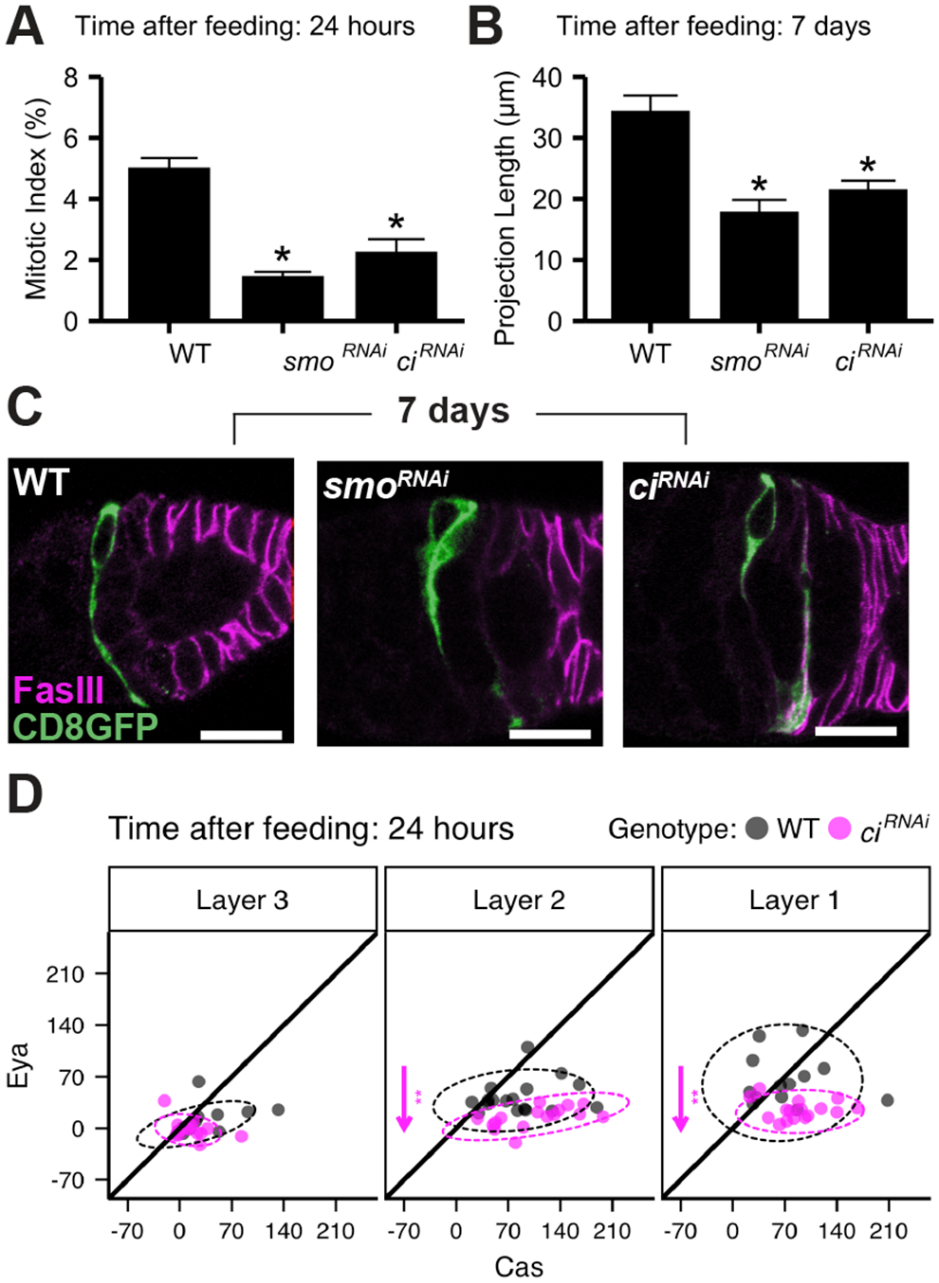
**Hedgehog effectors and actin regulators control FSC events during Q→P transitions.** (A) Layer 2 FSC mitotic index (PH3+ FSC/total) in WT (*109-30-Gal4 TubGal80^ts^*/+) versus RNAi knockdown (*109-30-Gal4 TubGal80^ts^*/*UAS-transgene*). *P<0.01 versus WT, χ^2^ test. Left to right: *n*= 538, 617, 322. Plot indicates mean→s.e.m. (B) Average FSC projection length 7 days after re-feeding; (*Ub-RFP, Gal80 FRT^19A^ Flp^122^*/*FRT^19A^*; *109-30 Gal4*/*UAS-transgene*). *P<0.01 versus *Ub-RFP, Gal80 FRT^19A^ Flp^122^*/*FRT^19A^*; *109-30 Gal4*/+, unpaired Mann–Whitney *U*-test. Left to right: *n*=6, 10, 9. Plot indicates mean±s.e.m. (C) CD8-GFP (green) marks FSCs and projections, with FasIII (magenta) (*Ub-RFP, Gal80 FRT^19A^ Flp^122^*/*FRT^19A^*; *109-30-Gal4*/*UAS-RNAi*). Scale bar: 10 μm. (D) MFI of FSC niche images 24 h after re-feeding [black (WT)=*109-30-Gal4TubGal80^ts^*/+; *Cas::GFP*/+, magenta=*109-30-Gal4 TubGal80^ts^*/*UAS-ci^RNAi^*; *Cas::GFP*/+]. Each dot represents the MFI of a single cell; ellipses represent 95% of the distribution. Arrows indicate significant [**P<0.01 (unpaired Mann–Whitney test with Benjamini Hochberg correction for multiple samples)] changes in Eya or Cas expression, pointing in the direction of change relative to WT. Left to right: *n*=17, 16.

Our next goal was to assess the potential role of FSC projections in establishment of differentiation patterning. Previously, we identified integrins as key regulators of projection growth and orientation (Hartman et al., 2015). The feeding-dependence of the projection growth response also suggested a potential role for Hh signaling in the process. Knockdown of the Hh effectors *smo* or *ci* abrogated both FSC proliferation (Fig. 5A) and feeding-responsive projection growth in FSCs (Fig. 5B,C), and Eya levels were significantly reduced in Layers 2 and 1 upon *ci^KD^* (Fig. 5D). The role of Hh at the top of the feeding-responsive signaling response and the resulting pleiotropic phenotypes emphasize the need for a genetic intervention that affects projection growth without blocking earlier events such as proliferation. To identify this tool, we took two approaches. First, we tested candidate genes with two key features: (1) known drivers of cytoskeleton arrangement, that (2) act downstream of Smo to mediate Hh signaling (Drummond et al., 2018; Gallo, 2011; Sasaki et al., 2010). Whereas altered activity of some candidates, including the small GTPase *Cdc42* and the actin regulator *Arp2,* mimicked integrin or Hh pathway proliferation and projection growth defects (Fig. S1A-D), others had no effect (Fig. S1A). Cdc42/Arp2 may function to mediate integrin signaling (Etienne-Manneville, 2004) or modulate Hh signaling directly (Drummond et al., 2018; Wan et al., 2013), acting high in the hierarchy to influence multiple downstream effects of feeding-dependent signaling.

**Fig. 6.**
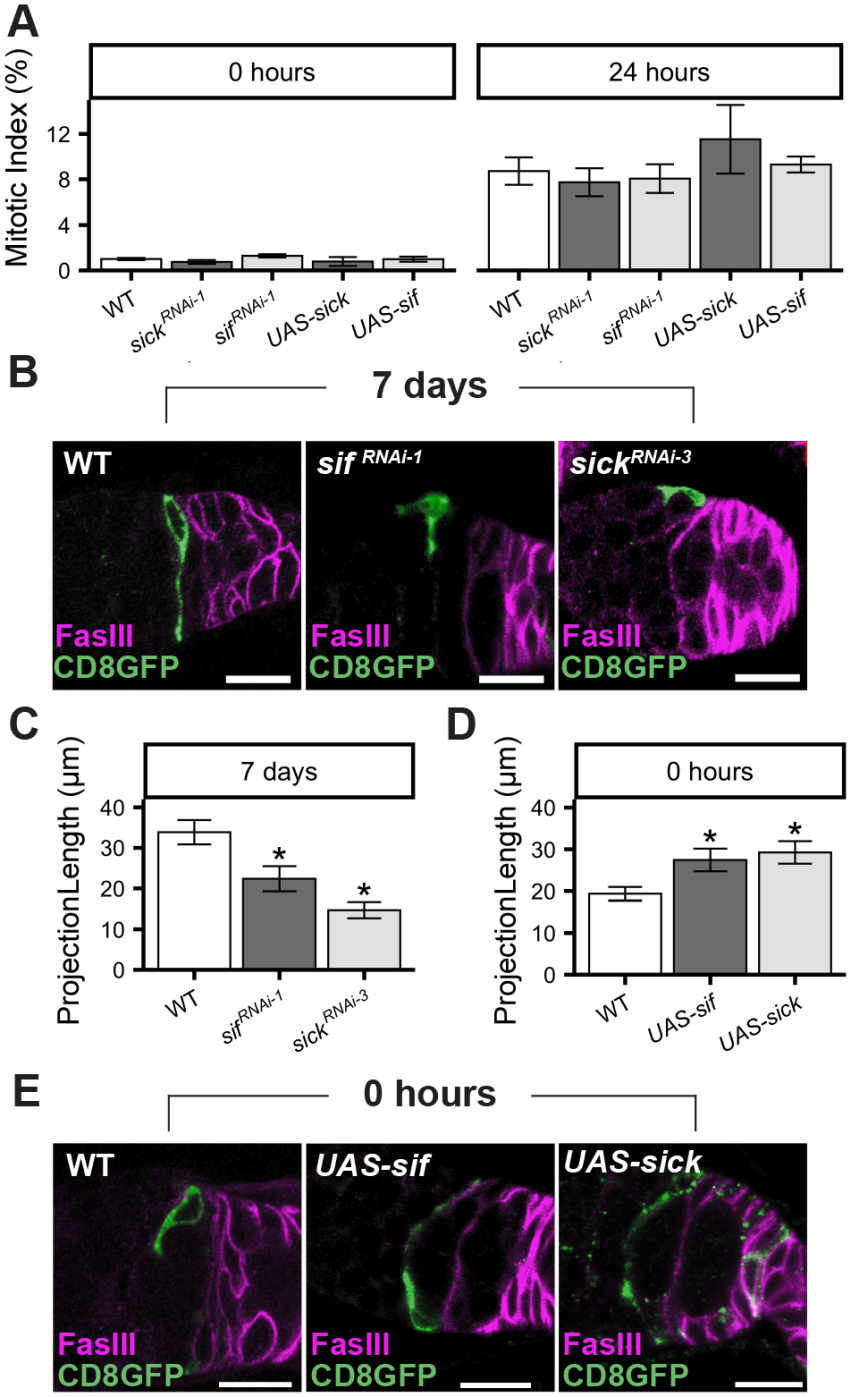
***still life* and *sickie* control feeding-dependent growth of FSC projections but not proliferation during Q→P transitions.** (A) Mitotic index (PH3+/total germaria) of Layer 2 FSCs (*109-30-Gal4*/+; *UAS-transgene*/+) 0 h (left) or 24 h (right) after re-feeding. Left to right: *n*= 606, 555, 682, 252, 514, 553, 546, 375, 285, 306. No statistically significant differences were observed (χ^2^ test). (B) CD8-GFP (green) marks FSCs and projections 7 days after re-feeding, with FasIII (magenta). (*Ub-RFP, Gal80 FRT^19A^ Flp^122^*/*FRT^19A^*; *109-30-Gal4*/*UAS-transgene*). (C,D) Average FSC projection length 7 days (C) and 0 h (D) after re-feeding. *P<0.01 versus *Ub-RFP, Gal80 FRT^19A^ Flp^122^*/*FRT^19A^*; *109-30-Gal4*/+, unpaired Mann–Whitney *U*-test. Left to right: (C) *n*=6, 9, 7; *N*=3; (D) *n*=9, 10, 12; *N*=3. (E) CD8-GFP (green) marks FSCs and projections during nutrient-restriction, with FasIII (magenta). (*Ub-RFP, Gal80 FRT^19A^ Flp^122^*/*FRT^19A^*; *109-30-Gal4*/*UAS-transgene*) (B,E) Scale bars: 10 μm. *sick^RNAi-1^*=*sick^HMJ21480^; sick^RNAi-3^*=*sick^HMC03544^; sif^RNAi-1^*=*sif^JF01795^.* Plots represent mean±s.e.m.

Unlike candidates with pleiotropic effects, reduced expression of *still life* (*sif*), the fly homolog of the guanine nucleotide exchange factor TIAM-1 (Sone et al., 1997), specifically affected projections. *sif^KD^* had no effect on feeding-induced proliferation 24 h after re-feeding (Fig. 6A), but blocked FSC projection growth (Fig. 6B,C). TIAM-1 binds directly to Smo in mammals and is known to activate downstream pathways to control neuronal protrusion, neurite extension, and axon guidance (Demarco et al., 2012; Kunda et al., 2001; Mertens et al., 2006; Ng and Luo, 2004; Sasaki et al., 2010; Sone et al., 1997; Zheng et al., 2016), processes with appealing similarities to FSC projection growth.

### *sickie* and *still life* are necessary and sufficient for FSC projection regulation

In addition to screening Smo effectors, we cloned the gene associated with the *109-30-Gal4* driver. *109-30*-Gal4 activates expression of genes under UAS control, with specificity for FSCs and their immediate progeny (Hartman et al., 2010) (Fig. 3A). This robust and useful expression pattern suggested that the associated gene likely was expressed and possibly functional in FSCs. Using Splinkerette PCR (Potter and Luo, 2010), a 500 bp band of genomic DNA was isolated from *109-30-Gal4* flies, matching the insertion locus (Fig. S2). Sequencing revealed that *109-30-Gal4* is inserted in the *sickie* (*sick*) gene, a known regulator of axon growth in mammals, worms, and flies (Abe et al., 2014; Coy et al., 2002; Maes et al., 2002; Merrill et al., 2002; Schmidt et al., 2009). A second *Gal4* insertion*, sick^MI08398-TG4.0^*, revealed the same pattern of expression (Fig. S3), and the lethal allele, *sick^NP0608^*, failed to complement *109-30-Gal4*, confirming the identity of *109-30-Gal4* as *sick-Gal4*. Notably, *sick* signals downstream of *sif* to control axonal outgrowth (Ng and Luo, 2004; Zheng et al., 2016). Similar to the effects of *sif* on FSC projection growth, *sick^KD^* in FSCs resulted in short, thickened projections (Fig. 6B,C). Proliferation during the 24 h timecourse was not affected by *sick^KD^* (Fig. 6A) or overexpression of *sick* or *sif* (Fig. 6A), emphasizing the separation of proliferation and projection growth during the Q→P transition. Importantly, *sick* and *sif* were sufficient to drive projection growth in nutrient-restricted flies, with overexpression of either gene increasing projection length under nutrient restriction conditions (Fig. 6D,E).

### *sif* and *sick* control differentiation patterning

**Fig. 7.**
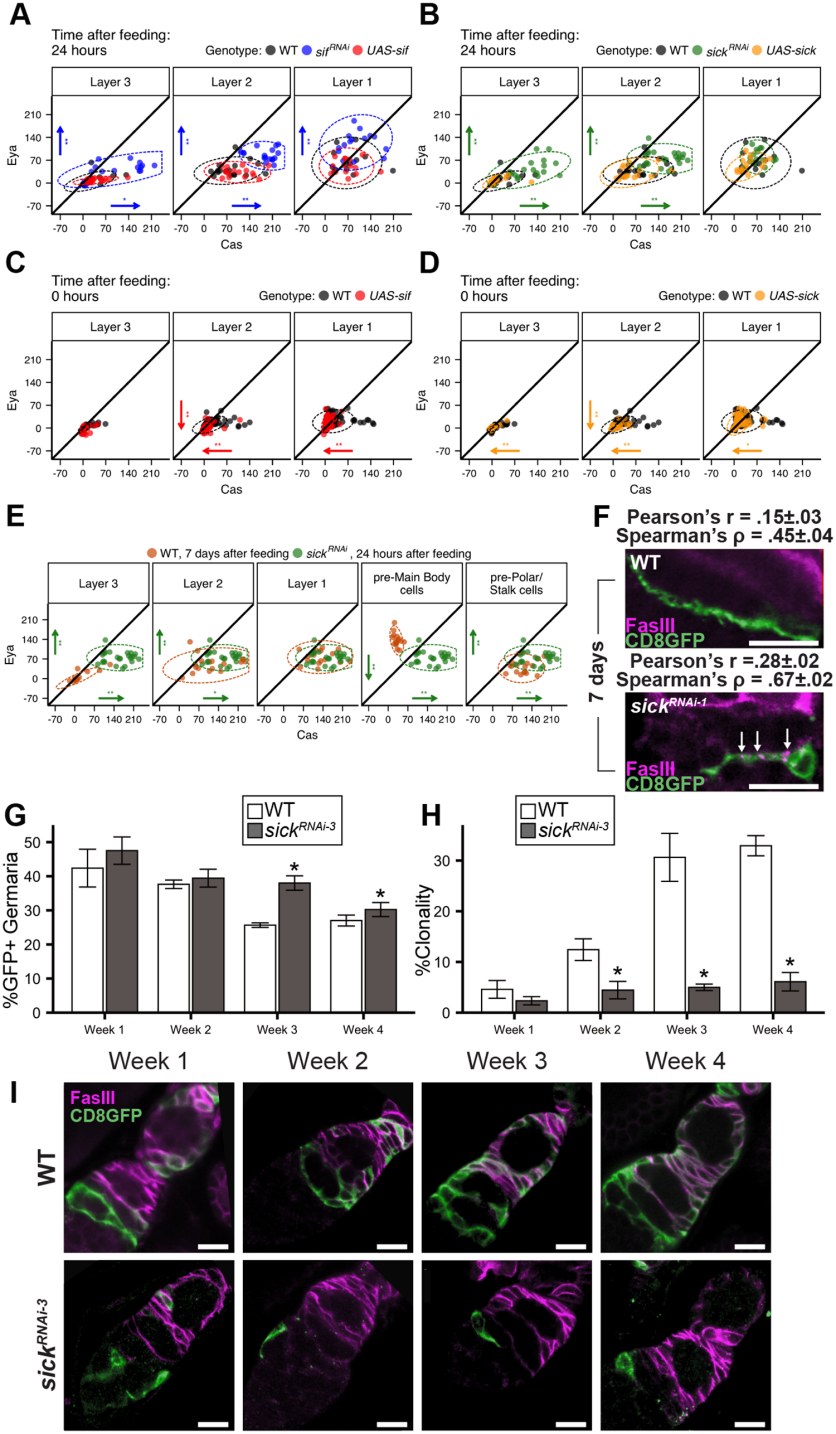
***sif* and *sick* are necessary and sufficient to control Eya-Cas expression in response to feeding.** (A-D) MFI of indicated cells 24 h after re-feeding (A,B) or at 0 h (C,D) [black (WT)=*109-30-Gal4TubGal80^ts^*/+; *Cas::GFP*/+, (other colors)=*109-30-Gal4TubGal80^ts^*/*UAS-transgene*; *Cas::GFP*/+]. Each dot represents the MFI of a single cell; ellipses represent 95% of the distribution. Arrows indicate significant (*P<0.05,**P<0.01, unpaired Mann–Whitney test with Benjamini Hochberg correction for multiple samples) changes in Eya or Cas expression, pointing in the direction of change relative to WT. (A) WT (*n*=17) versus *sif^RNAi^* (*n*=17) versus *UAS-sif* (*n*=16) at 24 h. (B) WT (*n*=17) versus *sick^RNAi^* (*n*=25) versus *UAS-sick* (*n*=18) at 24 h. (C) WT (*n*=62) versus *UAS-sif* (*n*=56) at 0 h. (D) WT (*n*=62) versus *UAS-sick* (*n*=56) at 0 h. (E) 7 days after re-feeding, WT (orange, *n*=16) versus *sick^RNAi^* Layer 2 FSCs (green, *n*=25) 24 h after re-feeding. (F) Co-localization (arrows) of CD8-GFP-labeled FSC projections (green) and FasIII (magenta)*^i^*. Pearson's and Spearman's correlation coefficients range from −1 to 1, with 1=complete colocalization and 0=absence of correlation. Both metrics show increased colocalization in *sick^RNAi^* (*n*=10) projections relative to WT (*n*=10) (*Ub-RFP, Gal80 FRT^19A^ Flp^122^*/*FRT^19A^*; *109-30-Gal4*/+). (G) % germaria bearing WT or *sick^RNAi^* MARCM-labeled FSCs over a 4-week timecourse. *P<0.01 (χ^2^ test) versus WT (*109-30-Gal4/CD8-GFP*). Left to right: *n*=367, 559, 537, 460, 419, 475, 801, 1047. (H) % fully clonal germaria, with all FSC progeny GFP-labeled. *P<0.01 (χ^2^ test) versus control (*109-30-Gal4/CD8-GFP*). Left to right: *n*=160, 265, 204, 183, 107, 181, 194, 316. (I) CD8-GFP-labeled FSCs (green) and follicle cells (magenta). (F,I) Scale bars: 10 μm.

The observation that *sif* and *sick* impact projection growth without affecting proliferation during the first 24 h after re-feeding afforded the opportunity to investigate the effect of projections on differentiation patterning, absent any loss of proliferative capacity. At 24 h post-feeding, we found *sif^KD^* or *sick^KD^* dramatically increased Cas and Eya in Layer 2 FSCs (Fig. 7A,B), consistent with adoption of a ‘more differentiated phenotype’. In Layer 3, Eya-Cas expression reached levels similar to those observed in wild-type Layer 2 cells, indicating a shift toward a more posterior cell fate upon *sif^KD^* or *sick^KD^*. Overexpression of either gene had no effect on Eya-Cas levels 24 h after re-feeding (Fig. 7A,B), when FSC projections are fully extended (Figs 1B and 4C). Conversely, overexpression of *sif* or *sick* in nutrient-restricted FSCs promoted projection growth in the absence of proliferation (Fig. 6A,D), reducing Eya-Cas levels in Layer 2 FSCs (Fig. 7C,D). Cas levels dropped in nutrient-restricted Layer 1 cells, further shifting the Eya-Cas signature towards a more anterior cell fate (Dai et al., 2017) (Fig. 7C,D). Together, these results are consistent with roles for *sif* and *sick* in promoting plasticity or suppressing differentiation via regulation of Eya-Cas expression.

During egg chamber formation, Layer 1 cells differentiate into pre-MB (Eya^hi^, Cas^lo^), or pre-PS (Eya^lo^, Cas^hi^) cells (Fig. 2) (Bai and Montell, 2002; Chang et al., 2013; Dai et al., 2017). This cell fate decision is also characterized by upregulation of the polarity protein FasIII, which is expressed in all follicle cell precursors early in development, and remains a definitive marker of polar and stalk cells throughout oogenesis (Fig. 1A) (Bai and Montell, 2002; Ruohola et al., 1991; Zhang and Kalderon, 2000). Eya-Cas patterns in *sick^KD^* Layer 2 FSCs 24 h after re-feeding were indistinguishable from a steady-state Layer 1 signature, and exhibited even higher expression of Eya and Cas relative to pre-PS cells (Fig. 7E). The Layer 2 signature in *sick^KD^* FSCs was most similar to pre-PS cells, with high expression of Cas as a key characteristic (Fig. 7E). Consistent with the possibility that these cells are precociously differentiated, *sick^KD^* FSCs aberrantly upregulated FasIII, with strong FasIII puncta along FSC projections (Fig. 7F). These FSCs were retained in the niche at rates similar to wild-type during the first 2 weeks after re-feeding, with unexpectedly higher retention of *sick^KD^* FSCs at later timepoints (weeks 3 and 4, Fig. 7G,I). Despite enhanced ability to remain in the niche, *sick^KD^* FSCs were unable to produce functional daughters (Fig. 7H,I).

## DISCUSSION

Q→P transitions are important for long-term stem cell retention in many systems. The balance between quiescent and proliferative states is critical for ensuring sufficient production of differentiated daughters needed for long-term tissue function. Q→P transitions also prevent accelerated aging via exhaustion of the stem cell pool or, conversely, tumorigenesis (Urbán and Cheung, 2021). Challenges with isolating and visualizing stem cell behavior during this critical time, as well as heterogeneity within stem cell pools, have prevented major advances in defining sequential events that drive Q→P transitions and how they are interrelated (Urbán and Cheung, 2021). The ability to impose quiescence through nutrient restriction and stimulate the transition to proliferation by feeding (Hartman et al., 2013) makes *Drosophila* ovarian FSCs an ideal system for defining molecular regulation of cellular events during this important process. Here, we demonstrate that the Q→P transition occurs during the first 6 h after re-feeding (Fig. 1), providing a well-defined time window for assessment of specific mechanisms that regulate cell cycle entry and patterning. Following the Q→P transition, FSCs extend projections to form an interwoven web-like structure that spans the niche (Figs 1 and 4) (Hartman et al., 2015). Appropriate projection growth is necessary to regulate feeding-dependent induction of the differentiation factors Eya and Cas (Fig. 7). The patterns of proliferation, projection extension, and differentiation established within FSCs during the first 24 h after re-feeding are sustained beyond the transition period into steady-state (Figs 1 and 2), emphasizing the critical importance of Q→P transitions for establishment of patterns that promote homeostasis over the long term.

In most cases, FSCs undergoing differentiation leave the niche, integrating into the follicular epithelium as main body follicle cells (Eya^+^, Cas^−^) or polar/stalk cells (Eya^−^, Cas^+^) (Bai and Montell, 2002; Chang et al., 2013; Dai et al., 2017; Margolis and Spradling, 1995). FSCs with disrupted projections have a different outcome, as they (1) express differentiation markers, (2) remain in the niche and (3) are unable to produce follicle cell progeny (Fig. 7). The implications of precocious differentiation of cells residing within the stem cell niche may be broad, as accumulation of senescent or partially differentiated cells within the niche is a hallmark of aging (McHugh and Gil, 2018). Previous work in FSCs has shown induction of FasIII under conditions of elevated JAK-STAT or JNK signaling, with increased conversion of FSCs to follicle cells and reduced FSC numbers (Melamed and Kalderon, 2020). Under those conditions, cells aberrantly expressing FasIII organized as follicle cells, forming epithelial-like structures around germline cysts while remaining at the Region 2A/2B border (Melamed and Kalderon, 2020). *sick^KD^* increased FasIII expression (Fig. 7F), but cells remained in the niche, without the ability to either produce daughters or organize as follicle cells. Perhaps separating proliferation control from differentiation marker induction in *sif^KD^*or *sick^KD^* FSCs drives the distinction, as JAK-STAT and JNK impact both processes. Alternatively, *sif^KD^*or *sick^KD^* FSCs may be rendered quiescent or senescent, unable to interpret feeding-dependent signals to enable normal FSC function. Given the propensity of oncogenes to initially induce senescence (Bianchi-Smiraglia et al., 2017; Zhu et al., 2020) and the importance of quiescent states in cancer risk (Paul et al., 2022), it may be of high interest to understand the phenotype observed in *sif^KD^* and *sick^KD^* FSCs as a mechanistic target to prevent transformation.

We present evidence that feeding-dependent proliferation and induction of differentiation are separable events. We found that String-induced proliferation was unable to promote Eya-Cas expression in nutrient-restricted or fed conditions (Fig. 3), demonstrating that simply transitioning to proliferation is insufficient for Eya-Cas induction. Previous work implicates proliferation as a driver of self-renewal, with the most proliferative FSCs exhibiting competitive advantage for niche occupancy (Amoyel et al., 2014; de Navascués et al., 2012; Greulich and Simons, 2016; Hsu et al., 2017; Jin et al., 2008; Kronen et al., 2014; Reilein et al., 2018; Snippert et al., 2010; Su et al., 2018). Based on the propensity of highly proliferative FSCs to self-renew, one prediction might be that high proliferation rates should correlate with the Eya^+^, Cas^+^ signature associated with self-renewal (Dai et al., 2017). Along these lines, mutations in Wnt effectors affect both proliferation (Kim-Yip and Nystul, 2018; Melamed and Kalderon, 2020; Song and Xie, 2003; Wang et al., 2021) and Eya expression in FSCs (Dai et al., 2017), with precocious differentiation to an Eya^lo^, Cas^hi^ polar/stalk cell fate (Dai et al., 2017). By contrast, Hh pathway activation via mutation of the negative regulator, *ptc,* dramatically increases proliferation (Zhang and Kalderon, 2000), but maintains cells in an FSC-like state, with low, but equal Eya and Cas expression causing a differentiation delay (Dai et al., 2017). The differential outcomes of hyperproliferative mutants on Eya-Cas are consistent with independent control of proliferation and differentiation (Figs 3, 6 and 7). Perhaps Wnt and Hh (1) activate pathways that control each event via distinct effectors, (2) act temporally to sequentially activate transcriptional targets that control each event, and/or (3) integrate with temporally and spatially regulated signals that contribute to cell fate outcomes.

Although proliferation was insufficient to direct differentiation patterning, we found a striking dependence of Eya-Cas expression on projection length (Fig. 7). Stalled or misdirected projections prevalent in *sif^KD^*or *sick^KD^* FSCs (Fig. 6) shifted a self-renewing, stem cell signature (Eya^lo^, Cas^lo^) to a more differentiated state (Eya^+^, Cas^hi^), as well as induced aberrant FasIII expression (Fig. 7). Conversely, *sif* or *sick* overexpression induced projection growth in nutrient-restricted conditions, driving reduced Eya-Cas expression (Figs 6 and 7). This is consistent with a model in which full-length projections suppress differentiation to promote self-renewal. An exciting possibility is that signals transmitted via projections between FSCs activate pathways to prevent feeding-dependent upregulation of Eya-Cas and maintain a plastic state. *sif* and *sick* are best known as regulators of axon growth via control of Cofilin, an actin severing protein (Abe et al., 2014; Coy et al., 2002; Maes et al., 2002; Merrill et al., 2002; Ng and Luo, 2004; Schmidt et al., 2009). Actin dynamics involving polymerization (mediated by Cdc42/Arp2) and depolymerization (mediated by Cofilin) are essential for axonal growth (Dent et al., 2011; Flynn et al., 2012; Hall and Lalli, 2010; Ng and Luo, 2004). FSC projections and axons share multiple characteristics, including dependence on Sif-Sickie-Cofilin and Cdc42-Arp2 (Figs 4 and 6). The observation that *sif* or *sick* expression in nutrient-restricted FSCs drives projection outgrowth (Fig. 6) emphasizes that this pathway is necessary and sufficient for controlling this process. In fed flies, Hh-mediated Smo activation may lead to Sif recruitment and activation, a mechanism similar to activation of TIAM-1 in mammalian cells (Sasaki et al., 2010). A less likely possibility is that Ci may activate expression of genes needed for *sif* or *sick* function. We found that *ci* was required for full Eya induction after re-feeding (Fig. 5) rather than functioning as a suppressor like *smo* (Dai et al., 2017*)*, *sif*, or *sick* (Fig. 7), suggesting that *ci* promotes expression of genes required for differentiation rather than acting to suppress them. *sick* is highly enriched in FSCs (Jevitt et al., 2020; Rust et al., 2020; Slaidina et al., 2021), but its expression is neither feeding- nor *ci*-dependent (data not shown). Taken together, these results suggest that the primary mode of feeding-dependent patterning of FSC cell fate occurs post-transcriptionally. Alternatively, Hh signaling may initiate sequential events in response to feeding, with time-dependent input from multiple pathways promoting *sif*-*sick*-mediated projection outgrowth.

In addition to defining new roles for *sif* and *sick* in FSC dynamics, we provide insight into the temporal and spatial regulation of Eya-Cas during Q→P transitions and steady-state feeding. Nutrient restriction reduced Eya and Cas in Layers 2 and 1 to levels normally observed in Layer 3 IGS/escort cells (Fig. 2), potentially limiting their capacity to self-renew and/or differentiate. It is possible that this effectively equalizes cells in the three layers, providing a re-set in preparation for a tumultuous entry into proliferation upon re-feeding. Recent work demonstrated increased ability of Layer 3 IGS/escort cells to function as FSCs following a period of starvation (Reilein et al., 2017; Rust et al., 2020), perhaps enabled by equalized Eya-Cas signatures between cell layers. Eya-Cas patterns depended on *sif* and/or *sick* at the 24 h timepoint (Fig. 7), emphasizing the importance of these newly identified regulators in dictating cell fate decisions. Interestingly, forced *sif* and/or *sick* expression only affected Eya-Cas expression in nutrient-restricted conditions. We favor a model in which projection extension enables signaling between FSCs and/or germ cells to control self-renewal versus differentiation fate decisions.

Unexpectedly, nutrient-restriction had a strong effect on Eya-Cas in pre-MB and pre-PS cells undergoing differentiation. Whereas steady-state Eya-Cas patterns were established within 24 h in Layer 3 and 2 cells, substantial increases in Cas expression in particular occurred after the 24-h timepoint in cells that were further along the differentiation continuum (pre-MB, and pre-PS). These results emphasize the importance of rapid establishment of homeostatic cell fate patterning in Layer 2 FSCs and raise important questions regarding the impact of nutrient restriction on partially differentiated cells. Unambiguously positive effects of repeated Q→P transitions via caloric or nutrient restriction have been demonstrated for multiple stem cell populations (Mana et al., 2017). However, concern has been raised regarding impact on differentiated cells, particularly in the context of tumorigenesis and cancer progression (Clifton et al., 2021; Gross and Pears, 2021; Nowak et al., 2013; Wood et al., 2020). The fly germarium provides a new model for investigation of the effects of nutrient restriction cycles on both epithelial stem cells and their progeny, providing opportunities to delineate mechanisms that govern plasticity and differentiation status with broad implications.

## MATERIALS AND METHODS

### Animal model

#### Fly preparation

All fly stocks were raised on standard fly food (7.5 g/l agar, 83.6 g/l cornmeal, 50 ml/l molasses, 20 g/l yeast, 5.2 ml/l propionic acid, 10 ml tegosept/l). Nutrient restriction was accomplished by placing flies in collection cages on grape juice plates (50% grape juice, 1% acetic acid, 3% Bacto-Agar, 0.1% methylparaben in water; Correa et al., 2021) for a minimum of 3 days (Hartman et al., 2015). Note that molasses plates do not induce quiescence in FSCs (Ables et al., 2012; Hartman et al., 2013, 2015) and are thus not appropriate for nutrient restriction conditions needed to analyze Q→P transitions. Re-feeding during the 24-h timecourse was done by adding a water-based paste of baker's yeast in water to grape juice plates; 7-day timepoints were done by transferring 24-h re-fed flies to standard fly food for 6 additional days. Flies were maintained at standard 25°C, additional fly stocks were maintained at 18°C temperature-controlled incubators.

#### Fly strains and genetics

The following stocks were obtained from the Bloomington Drosophila Stock Center (BDSC, Bloomington, IN), *109-30-Gal4* (Hartman et al., 2010) [y^1^w*;P(GawB)109-30/CyO], *sick Trojan Gal4* [y^1^w*; Mi(Trojan-GAL4.0)sick^MI08398-TG4.0^/SM6a]*, smo RNAi* (Hartman et al., 2010) {y^1^w*;P[*w(+mC)=UAS-smo.RNAi*]} (Clevers and Watt, 2018; Goodell and Rando, 2015*;* Hsu et al., 2017) 2 *P(UAS-smo.RNAi*)*8/CyO, P(Wee-P.ph0*)*2*, *ci RNAi* (Singh et al., 2018) [*yv; P(TRiP.JF01715)attP2*], *UAS-string* (Singh et al., 2018) [*w^1118^; P(UAS-stg.N)4*], *cdc42* dominant negative [w*; P(UAS-Cdc42.L89)4], *arp2* RNAi [*y^1^v^1^; P(TRiP.JF02785)attP2/TM3, Sb^1^*], *Inx2* RNAi [*y^1^v^1^; P(TRiP.JF0244)attP2*]*, zpg* RNAi [*y^1^v^1^; P(TRiP.JF02753)attP2*], InR RNAi [*y^1^v^1^; P(TRiP.JF01482)attP2*]*, sif RNAi-1* [*y^1^v^1^; P(TRiP.JF01795)attP2*], *sick RNAi-1* {*y^1^v^1^; P(y)+t7.7* [*v(+t1.8)=TRiP.HMJ21480]attP40*}, *sick RNAi-3* [y^1^sc*v^1^sev^21^; P(TRiP.HMC03544)attP2], *UAS-sif* [w*; P(UAS-sif.S)M3.1], UAS-sif [w*; P(w+mC)=UAS-sif.S}M3.1], UAS-CD8-GFP (Ub-RFP, *Gal80 19AFRT Flp122*; *UAS-CD8-GFP), UAS-CD4-GFP* {P[y(+t7.7)w(+mC)=CoinFLP-LexA::GAD.GAL4]attP40,P[w(+mC)=lexAop-rCD2.RFP]2;P[w(+mC)=UAS-CD4-spGFP1-1]3,P[w(+mC)=lexAop-CD4-spGFP11]3/TM6C}. We also obtained stocks from the Kyoto Stock Center (DGRC, Kyoto, Japan), *UAS-sick* (y*w*; P[w+mC=UAS-sick.A]4844-1-8-M), *sick-Gal4*(w*;P[GawB]sick^NP0608^/CyO). *Cas::GFP* [*FlyFos020486*(*pRedFlp-Hgr*)(*CG1211826169::2XTY1-SGFP-V5-preTEV-BLRP-3XFLAG*)*dFRT*] was obtained from the Vienna Drosophila Resource Center (VDRC, Vienna, Austria).

### Dissections and immunostaining

#### Randomization

For all experiments, 15-20 flies of the indicated genotype were selected at random from a larger pool. Ovaries from ∼1-week-old adult female flies (*Drosophila melanogaster*) were dissected in Grace's insect cell culture medium (Gibco, Gaithersburg, MD, USA), fixed in 4% paraformaldehyde for 15 min and then washed three times in 1X PBST for 5 min. The ovaries were then incubated with primary antibodies in 0.5% normal goat serum diluted with 1X PBST solution overnight at 4°C. The ovaries were washed three times for 10 min each in 1X PBST and then incubated with secondary antibodies at RT for 1 h. Ovaries were washed three times in 1X PBST. The ovaries were then mounted on slides using Vectashield medium (Vector Laboratories, Burlingame, CA, USA).

#### Inclusion criteria

No animals were excluded from analysis in this study.

#### Reagents

Primary antibodies used were mouse anti-Fasciclin III (FasIII) (1:200; 7G10, DSHB, Iowa City, IA; Patel et al., 1987), mouse anti-Eya (eya10H6, 1:40, DSHB; Boyle et al., 1997), chicken anti-GFP (1:1000, Cat# PA1-9533, Thermo Fisher Scientific, Waltham, MA), rabbit anti-PH3 (1:1500, Cat# HO412, MilliporeSigma). All secondary antibodies used were Alexa antibodies conjugated to species-specific secondary antibodies (1:200; Thermo Fisher Scientific).

### Creating Mosaic clones in germaria

Mosaic analysis with repressible cell marker (MARCM) stocks were generated by crossing *Ub-RFP, Gal80 FRT^19A^ Flp^122^*/Y; *UAS*-*CD4-GFP or UAS-CD8-GFP*; UAS-transgene males to *FRT^19A^*; *109-30-Gal4*/CyO females (Hartman et al., 2015; Lee and Luo, 2001). Flies were heat shocked for 1 h at 37°C to obtain single clones of GFP positive labeled follicle stem cells. After the heat shock, female flies were kept at 25°C either in fly food vials or starved for on grape juice plates with males corresponding to different experimental designs. Fed flies were kept on fresh food sources for 3 days after heat shock before the ovaries were isolated. Germaria were stained with chicken anti-GFP and mouse anti-FasIII to image projections.

### Measurement of projection length

After images of single cell GFP-labeled FSCs were acquired in the MARCM-labeled stocks, projections of germarium images were imported into IMARIS for measurement. Multi-point length measurements were taken from the center of the cell nucleus to the end of the projection by using the measurement function in IMARIS. For the screening panel in Fig. S1, measurements were taken using Leica AF SP5 software.

#### Statistics

Significant differences in projection lengths were determined using unpaired Mann–Whitney *U*-tests, which assume a non-normal data distribution.

### FSC niche retention and clonality

MARCM stocks were generated as described above. Flies were heat shocked at 37°C for 1 h and placed in fresh vials subsequently at 25°C. Flies were flipped into fresh vials twice a week to ensure food availability. Ovaries were dissected and stained with chicken anti-GFP and mouse anti-FasIII at week 1, 2, 3 and 4 respectively. FSC niche retention was determined by scoring the percentage of germaria with GFP-positive clones in Region 2A/B. Functionality was determined by the presence of GFP-labeled FSC progeny in early stage egg chambers. Germaria with 100% of FSCs and follicle cells GFP-labeled were scored as fully clonal. Partial domination was not considered as clonal.

#### Statistics

For hypothesis testing, the number of GFP-positive and GFP-negative germaria were summed across biological replicates for each genotype. For each week, a χ^2^ test of independence was performed to determine correlation between genotype and FSC retention. A χ^2^ test of independence was also performed on GFP-positive germaria (fully clonal versus not clonal) to determine correlation between genotype and FSC clonality. *P*-values are reported with Yates correction.

### Proliferation assay

Flies were generated by crossing either *109-30-Gal4TubGal80^ts^*/CyO or *109-30-Gal4* to their corresponding UAS-transgene. Flies carrying *109-30-Gal4TubGal80^ts^*/UAS-transgene were incubated at 29°C prior to dissection. All samples were starved for 3 days prior to re-feeding with yeast for corresponding time points. Ovaries were dissected in Grace's insect medium and stained with rabbit anti-phospho-histone-H3 (PH3) and mouse anti-FasIII. After completing the immunofluorescence procedure described above, mitotic index was calculated as the number of germaria with at least one PH3-positive FSC, divided by the total number of germaria (Hartman et al., 2010; O'Reilly et al., 2008).

#### Statistics

Significant changes in mitotic index were determined by a χ^2^ test of independence.

### Quantification of Castor and Eya in FSCs

Confocal images were processed using ImageJ. All images were taken in the cross-section of the center of the germaria. FasIII expression was used to identify the germarium shape and FSC region. For each germarium, three regions of interest (ROI) were created that correspond to the three layers of FSCs in region 2A/2B, as described in Dai et al. (2017). These ROI were chosen based on FasIII expression. Additionally, a large ROI spanning the germline (where Cas-GFP and Eya are not expressed), was included for background subtraction. For wild-type samples, ROI corresponding to pre-Main Body cells and pre-Stalk/Polar cells were also included.

Signal intensity values from GFP (Cas) and Eya channels were extracted from each ROI, recorded along X–Y coordinates, and imported into R studio. Mean Fluorescence Intensity (MFI) was calculated by averaging across all X–Y coordinates in each ROI, then normalized by subtracting average background intensity of the appropriate background ROI.

#### Statistics

For hypothesis testing, MFI replicates were compared between experimental and control conditions for each channel, at each layer. *P-*values were determined from Mann–Whitney *U*-tests (which assume a non-normal data distribution) on these values, with a Benjamini-Hochberg correction for multiple testing.

Power analyses were performed to estimate the required *n* to confirm or reject the null hypothesis at *P*<0.05, based on effect sizes observed in the preliminary data.

### Co-localization of FasIII and GFP expression

Images of MARCM clones were analyzed by ImageJ. GFP-positive FSC projections were outlined as regions of interest by polygon selection. The Coloc2 plug-in was used to analyze GFP and FasIII co-localization. In some images, the brightness of the FasIII channel was enhanced to ensure visibility by altering Brightness/Contrast of the whole image using Adobe Photoshop.

#### Statistics

Pearson's correlation coefficients (R) and Spearman's rank correlation coefficients (ρ) were recorded and averaged between replicate images.

### Splinkerette PCR

Splinkerette PCR (Potter and Luo, 2010) was used to map the *pGawB-GAL4* insertion in *109-30-Gal4* flies. Genomic DNA was isolated (Promega, Madison, WI, USA) according to the manufacturer's protocol. Genomic DNA was digested by BstYI and ligated to Splinkerette oligonucleotides, followed by two rounds of PCR, exactly according to the published Splinkerette PCR protocol (Potter and Luo, 2010). The ∼500 bp DNA band was gel extracted (Qiagen, Germantown, MD, USA) and sequenced.

### Image analysis

Images were collected at room temperature using 40X (1.25 NA) or 20X (0.7 NA) oil immersion lenses (Leica) on an upright microscope (DM 5000; Leica Microsystems, Wetzlar, Germany) coupled to a confocal laser scanner (TCS SP5; Leica). LAS AF SP5 software (Leica) was used for data acquisition. Images representing individual channels of single confocal slices or three-dimensional reconstructions of the germarium, including the FSC region were exported into IMARIS or Fiji (ImageJ) for further analysis.

Image acquisition and data analysis were conducted objectively, with investigators agnostic to the outcome of the experiment. Outcomes were determined after a thorough analysis of the data by a separate investigator.

## Supplementary Material

10.1242/biolopen.059625_sup1 Supplementary information
