# Supplementary material for: Sequential events during the quiescence to proliferation transition establish patterns of follicle cell differentiation in the Drosophila ovary
Source: Biol Open. 2023 Jan 12;12(1):bio059625. doi: 10.1242/bio.059625 (PMC9867896; doi:10.1242/bio.059625)
Supplement: Supplementary information [file biolopen-12-059625-s1.pdf]

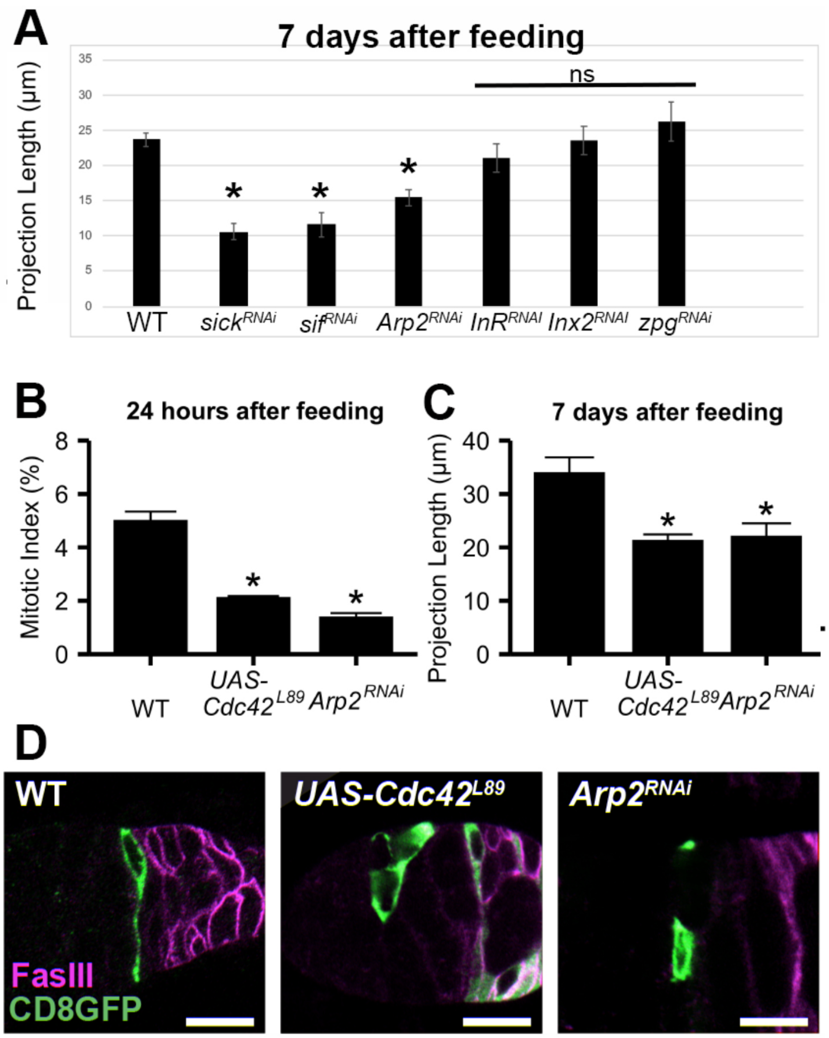

Fig. S1. arp2 and cdc42 are required for proliferation and projection growth. (A) Screen for regulators of projection length: average FSC projection length 7 days after re-feeding is indicated; (Ub-RFP, Gal80 FRT19A Flp122/FRT19A; 109-30-Gal4/UAS-transgene). *p<0.0001 vs. Ub-RFP, Gal80 FRT19A Flp122/FRT19A; 109-30-Gal4/+. Left to right: n= 12, 14, 9, 8, 4, 4. B) Layer 2 FSC mitotic index (PH3+ FSC/total) in WT (109-30-Gal4TubGal80ts/+) versus RNAi knockdown or ectopic gene expression (109-30-Gal4 TubGal80ts/UAS-transgene). *p<0.01 vs. 109-30-TubGal80ts/+. Left to right: n= 538, 477, 357. (C) Average FSC projection length 7 days after re-feeding; (Ub-RFP, Gal80 FRT19A Flp122/FRT19A; 109-30-Gal4/UAS-transgene). *p<0.01 vs. Ub-RFP, Gal80 FRT19A Flp122/FRT19A; 109-30-Gal4/+. Left to right: n= 6, 6, 8. (D) CD8-GFP (green) marks FSCs and projections. FasIII (magenta) marks follicle cells (Ub-RFP, Gal80 FRT19A Flp122/ FRT19A; 109-30-Gal4/UAS-transgene. Scale bars =10 μm. Plots indicate mean +/- SEM.

Biology Open • Supplementary information

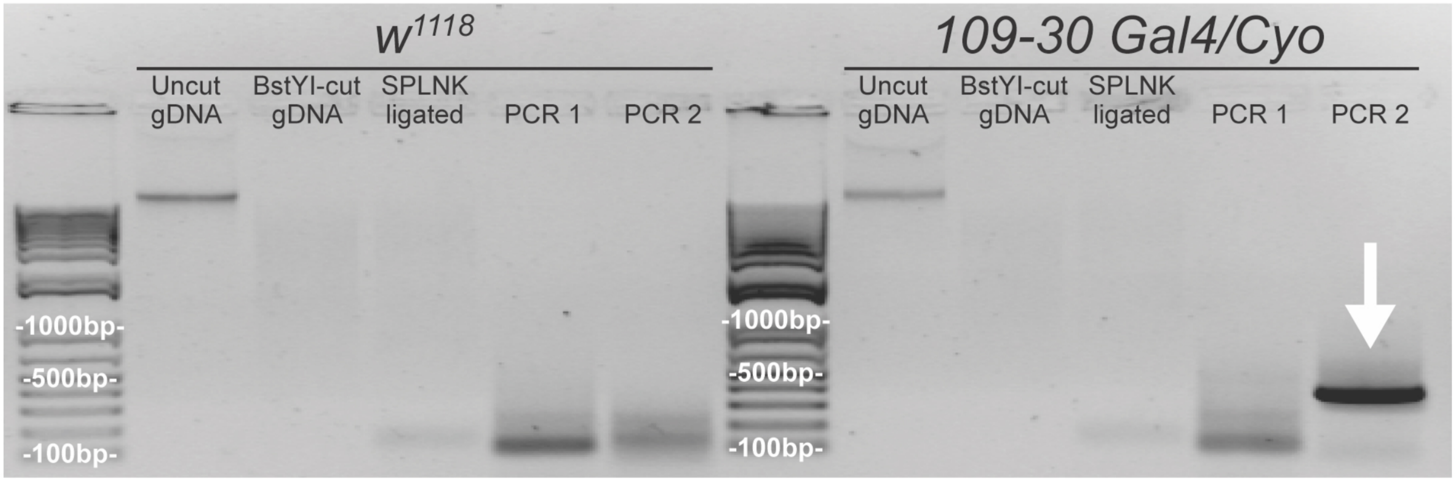

Fig. S2. ***109-30 Gal4 is sickie*.** Splinkerette PCR rescued a 500bp fragment (white arrow) from *109-30 Gal4* flies, but not w^{1118} flies, which lack a *Gal4* insertion. Sequencing revealed insertion in the sickie locus.

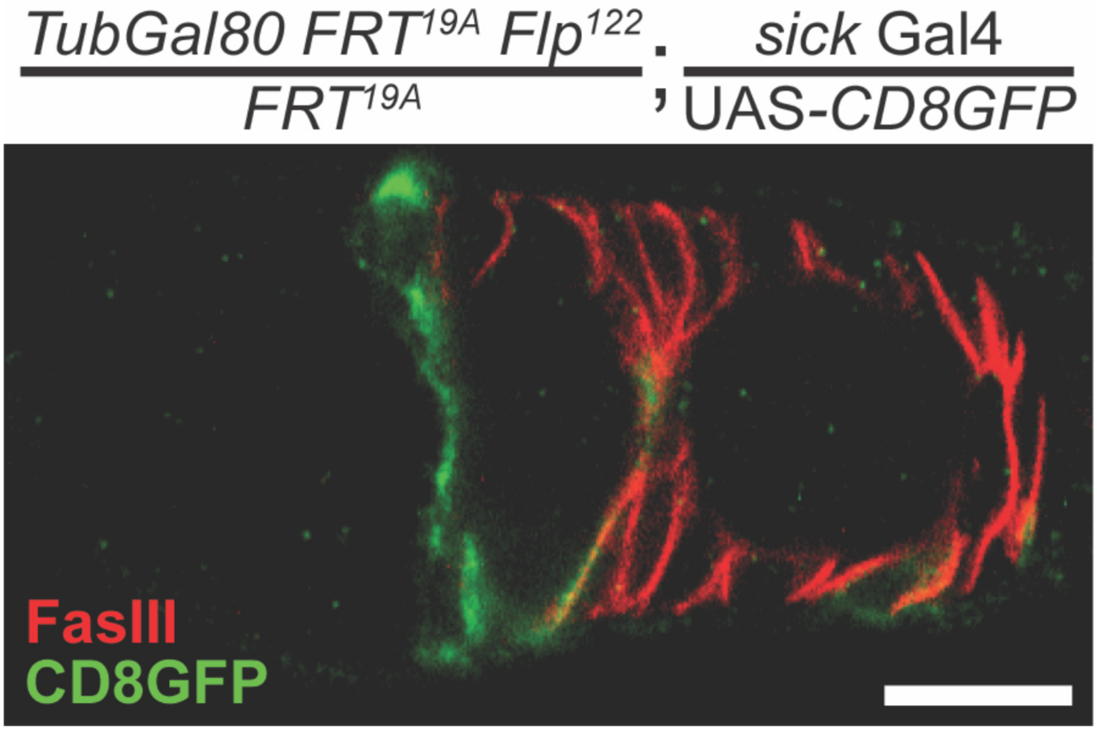

Fig. S3. *sick Gal4* drives expression in FSC and progeny. $sick^{MI08398\text{-}TG4.0}$ drives CD8GFP (green) expression in the *109-30 Gal4* pattern. FasIII (red) marks follicle cells. Scale bar is 10 μm.
